# CCEO-DCABNet: Chronological Chaotic Evolution Optimization-Enabled Hybrid Deep Learning for Multiclass Disease Classification Using Chest X-Ray Images in Federated Learning

**DOI:** 10.3390/diagnostics16132096

**Published:** 2026-07-03

**Authors:** Leena Patil, Bindu Garg, Massimo Donelli, Achin Jain

**Affiliations:** 1 Bharati Vidyapeeth (Deemed to be) University, College of Engineering, Pune 411043, India; 2 Department of Computer Science and Engineering, Walchand College of Engineering, Sangli 416415, India; 3 Department of Civil, Environmental and Mechanical Engineering, University of Trento, 38123 Trento, Italy; 4 Department of Information Technology, Bharati Vidyapeeth’s College of Engineering, New Delhi 110063, India; 5 INTI International University, Persiaran Perdana BBN Putra Nilai, Nilai 71800, Negeri Sembilan, Malaysia

**Keywords:** chest X-ray image, federated learning, MMPU-Net, Gaussian filter, chaotic evolution optimization

## Abstract

**Background:** Chest X-ray imaging is a widely used diagnostic modality for identifying various lung diseases. Accurate multiclass classification of lung diseases enables timely treatment and improves patient survival. However, disease detection using chest X-ray images remains challenging due to heterogeneous data, overlapping radiographic features, and data privacy concerns. Furthermore, distinguishing among different lung diseases is difficult because of their similar clinical manifestations and imaging characteristics. **Method:** To address these challenges, a novel chaotic evolution optimization-enabled deep channel-attention broad convolutional neural network (CCEO-DCABNet) is proposed for multiclass lung disease classification within a federated learning (FL) framework. The proposed model ensures enhanced data privacy by allowing multiple client nodes and a central server to collaboratively train the model without sharing raw data. Prior to classification, image preprocessing is performed using Gaussian filter-based denoising followed by multiscale unsharp masking-based image sharpening. Subsequently, multiclass disease classification is carried out using DCABNet, whose parameters are optimized through the proposed CCEO algorithm. In addition, the federated learning process employs an averaging strategy for local model updates and global aggregation. **Results:** The proposed CCEO-DCABNet achieves an accuracy, true positive rate (TPR), and true negative rate (TNR) of 96.98%, 96.41%, and 97.45%. **Conclusions:** Experimental results demonstrate that the proposed CCEO-DCABNet framework effectively classifies multiple lung diseases from chest X-ray images while preserving data privacy through federated learning. The model achieves superior classification performance and can support reliable computer-aided diagnosis in clinical settings.

## 1. Introduction

Chest X-ray uses minor quantity of ionized radiotherapy to produce images (grayscale) of the inner chest. Only physicians and radiologists can assess these images for viewing abnormalities in the chest. This test is needed to detect any kind of abnormalities in the heart, chest wall, and lungs, and it is also employed for diagnosing different symptoms (cough, breathing problems, and chest pain). It also supports analysis and treatment of different classes of lung illnesses. Due to its easy and fast computing capability, Chest X-ray images are helpful in emergency conditions [1]. These types of X-rays are employed as an affordable diagnostic model in medical imaging, and these images utilize less time to provide a rapid diagnosis. As a result, it can be utilized to triage patients for treatment planning and allocation of medical resources [2]. Chest X-ray images are usually created by projecting X-rays through the body and onto metallic plate of the X-ray equipment. These images use and non-invasive approach and an effortless medical test, in which an electric device emits radiation to the patient’s body to generate 2-dimensional (2D) view of the inner body’s structures. It is projected that around 3.5 billion analytical X-rays are processed worldwide, which accounts for 40% of the global whole images [3]. Different models with chest X-ray analysis are employed for detecting lung infections. Also, Computer-aided solutions are used to sense lung infection by examining chest X-ray images, and are used to assist fast diagnosis [4].

Chest X-ray is used as a diagnostic approach to assess thoracic infections, such as pneumonia, COVID-19, and pleural effusion [2]. Lung illnesses have a widespread impact on people and are prime causes of the global mortality rate. Moreover, illnesses, like chronic bronchitis, emphysema, pneumonia, and pulmonary fibrosis, have been related to an increased chance of developing lung illness. However, diagnosis of lung infection using X-ray images remains a complex for radio therapist. diagnosis approaches depend on manual interpretation by radiologists, which takes more time consumption. Several researchers are interested in developing automated lung disease detection systems for early detection. Hence, a computer-aided diagnostic (CAD) system is utilized to perform large-scale analysis of lung disease by assessing X-ray images [5]. With developments in information systems, the deep learning (DL), arithmetical examination, and machine learning (ML) models are used to identify different medical issues, improve clinical forecasts, and explain complicated features. These methods solved several computer vision problems in the medical field. DL is a subsection of ML that has offered a lot of interest due to its better performance in prediction and classification [6,7]. It offers good outcomes, but it needs a huge number of hyper parameters to process large datasets. The speedy growth of automatic DL-based image diagnostic methods enabled expert performance in specific tasks [8,9]. With substantial advances in DL and computer vision, computer-aided analysis offers an effective diagnostic process and lessens clinicians’ burden. Chest X-ray-based image analysis utilizing DL offers simple and consistent treatment for different types of lung illness [3]. DL model improves accuracy of predictions, and utilizes a variety of nonlinear functions for getting a reliable outcome [10]. DL, mainly convolutional neural networks (CNNs), is employed as an effective method to analyze medical imaging, because it extracts complex features from imaging data and allows them to learn patterns that are not discernible by humans. Application of Chest X-ray in lung disease classification offers a promising result in terms of human error reduction, clinical decision-making, and diagnostic precision [11]. Though DL-enabled chest X-ray image diagnosis for respiratory illnesses has shown cost-effectiveness and better accuracy, they do not perform well with imaging data from different sources. The utilization of FL in healthcare system offers better learning from different kinds of decentralized data and aggregating feature details from distributed edge nodes [12]. Also, FL is a type of distributed collaborative paradigm for coordinating the training of data from different sources. FL also guarantees medical services without relating to private data of individuals [13]. The most crucial aspect of FL is its distributed capacity, which retains data privacy from source to training. It also ensures user data secrecy, while the server attains local models from every client and generates a global model by aggregation. Hence, FL-based disease classification protects patient privacy and increases model generalization by combining data from numerous sources [14]. Classification of multiclass disease is done by DCABNet, and CCEO trains its parameters. The average method performs local updation and aggregation. Although each component like federated learning (FL), radiomics, attention techniques, and capsule models, is individually well-versed in medical imaging processing, their separate use alone is rarely capable of capturing the intricacies of chest diseases. Model concatenation will inevitably lead to overlapping of feature maps and higher computation costs. Our work in this paper is certainly not about just putting all those modules together into some sort of random combination. On the contrary, we create an interrelated network wherein chaotic evolutionary optimization serves to fill the void between statistical radiomic feature extraction and deep spatial abstraction. Through solving the problem of feature extraction, attention-based weights, and spatial routing in a unified manner, we generate a highly informative feature space.

This paper is structured into five distinct sections, beginning with an Introduction that establishes the motivation and contributions, followed by a Literature Survey reviewing existing multiclass classification methods. Section 3 details the core methodology, covering the federated learning framework, MMPU-Net segmentation, and the proposed CCEO-DCABNet architecture, while Section 4 presents a comprehensive evaluation of results, ablation studies, and comparative analysis. Finally, Section 5 concludes the study by summarizing findings, addressing clinical considerations, and proposing future research directions.

## 2. Literature Survey

G. Divya Deepak, Subraya Krishna Bhat [15] devised a multi-stage CNN-enabled classification of lung disease. It extracted discriminative attributes from different disease types. Nevertheless, generalizability and interpretability of this model were limited in a wider clinical setting. Reddy, K.D., Patil, A [16] developed chest X-ray (CXR)-MultiTaskNet-based joint disease identification and classification. This method yielded clinically valuable, scalable, and explainable output to improve decision-making. Still, it failed to incorporate a new explainability approach to attain better semantic reasoning and dependability. T. Geroski et al. [17] devised a SoftLungX-based respiratory illness classification. It minimized the requirement for wider data related to weights of the network and exploiting diagnostic efficiency. However, it failed to consider different metrics to evaluate the model’s performance. X. Fu et al. [18] devised LungMaxViT for classifying multi-class lung illness. In the method, CNN with an explainable transformer solved issues related to inter-class resemblances and variances of images. Nonetheless, it failed to consider fine-tuned models to increase the classification accuracy.

A. N. Patel et al. [19] developed EfficientNet-B4-based transfer learning (TL) in lung illness classification. This model addressed class imbalance problems and increased diversity of data resources. However, it failed to integrate with a real-world clinical setting for improving diagnostic accuracy. S. Ashwini et al. [20] devised a CNN with grid search optimization (CNN–GSO) to classify lung illness. It offered superior generalization and was employed as a reliable diagnostic approach in clinical settings. Nevertheless, it failed to solve privacy and overfitting issues across different imaging conditions. A. Makkar and K. Santosh [13] developed a secure aggregation method named SecureFed for lung illness classification. It offered superior privacy preservation against malicious updates and data leakage, and it improved robustness across heterogeneous datasets. Still, increased computational and communication overhead to degrade its efficiency. S. Durga et al. [21] devised a federated learning-based ensemble model (FLEM) for lung disease diagnosis. It minimized bandwidth utilization by distributing model updates, and interpretability was improved by XAI techniques. However, longer convergence time and model coordination due to communication overhead reduced the classification rate. Furthermore, in the distributed learning landscape, advanced frameworks like personalised heterogeneous human-centric federated learning (PHH-FL) have introduced methods to mitigate data non-IID constraints by balancing clinical client variance [22].

### Challenges

Difficulties related to existing multiclass disease classification methods are given by

- In [15] a multi-stage CNN achieved comprehensive lung illness classification with better predictive accuracy. Still, it did not combine multi-level convolutional and attention functions to improve segmentation performance.
- In [16], CXR-MultiTaskNet was employed as a unified DL for localizing joint disease. Still, this approach struggled due to intensity variations in the smoothing pixel and poor robustness against imaging features.
- SoftLungX in [17] classified chest X-ray images into radiological findings and disease categories with quick processing. Nonetheless, it failed to localize pictorial depictions for classifying and measuring disease severity
- LungMaxViT in [18] incorporated a multi-axis transformer with a CNN backbone for improving robustness of classification. Although this model captured both local and global features, it failed to analyze the osteoporosis condition.
- Classification of lung disease automatically detects and categorizes respiratory illnesses, like tuberculosis, pneumonia, lung cancer, and other illnesses. Detection and classification of disease improve treatment planning as per disease types. Due to class imbalance, limited annotated data, interpretability issues, and inconsistency in imaging quality, the detection of multiclass disease becomes complex.

## 3. System Model for Federated Assessment Using Chest X-Ray Images

**Figure 1:**
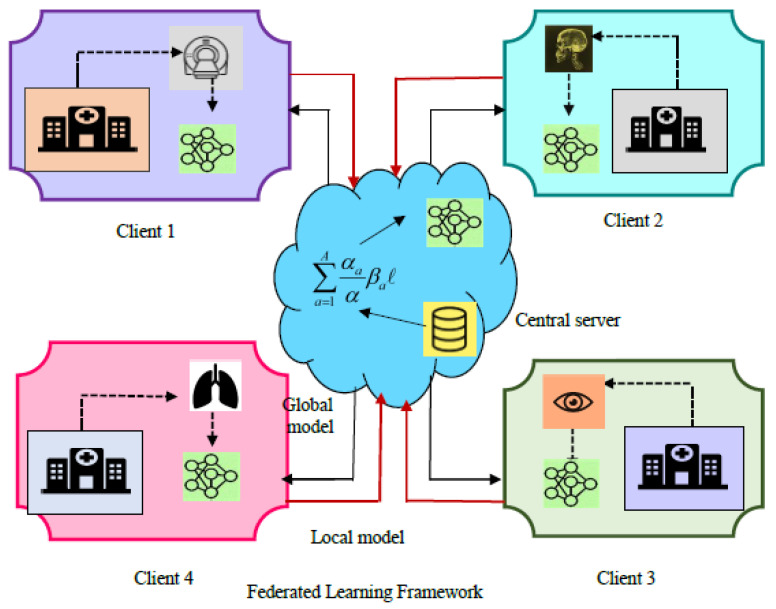
System model for federated learning in medical analysis.

The system model for federated learning in medical analysis [23] is given in Figure 1. Here, all clients are responsible for a particular task, and they gather medical records from different medical centers to compute several parameters. Afterward, the server aggregates these parameters for computing the weighted average and developing a global model. The global model is updated periodically and transmitted back to clients, and then the central server ensures privacy. The quantity of data across different clusters varies. Based on FedAvg, each client sends learning parameters to the server. Here, the server is aggregated by the averaging method, and mathematical form of FedAvg becomes,

(1) $$fun\left(\ell \right)\;=\;\sum_{a=1}^{A}\frac{\alpha_{a}}{\alpha }\beta_{a}\ell$$

Here, α count of clients is presented and $\beta_{a}\ell$ portrays the loss function. Weight of every loss with dimension of client’s dataset is specified by $\alpha_{a}$.

### 3.1. Chronological Chaotic Evolution Optimization-Enabled Deep Channel-Attention Broad Convolutional Neural Network

**Figure 2:**
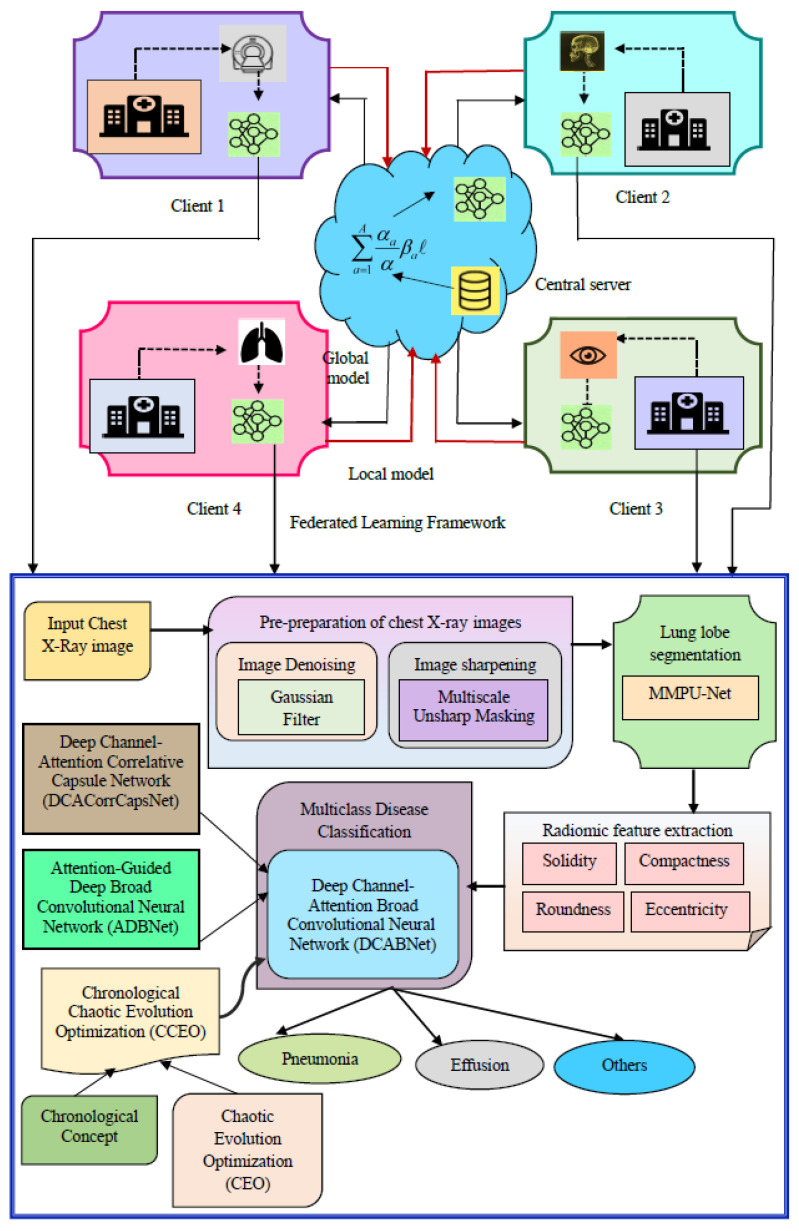
Pictorial depiction of CCEO-DCABNet-enabled for multiclass disease classification using FL.

Disease classification based on chest X-ray is needed for analyzing different lung diseases. Still, centralized data collection, aggregation, and limited generalization owing to heterogeneity impact disease classification. Class imbalance issues also impact the performance of multiclass disease classification. To overcome such challenges, a hybrid CCEO-DCABNet model using FL is introduced in this research. Local training at each node is done using local data, and trained data is updated on the server. Data is aggregated on the server and downloads global models. Afterward, the training model is updated, and iterated till maximum epochs. In the training model, the following processes are carried out. Initially, input is acquired from datasets [24,25], and then pre-preparation is performed. Here, denoising and sharpening are done using a Gaussian filter [26] and multiscale unsharp masking [27]. Segmentation of the lung lobe is done by MMPU-Net [28], and radiomic features [29] are extracted in the feature extraction stage. Further, lung diseases are categorized as effusion, pneumonia, and other by DCABNet. Here, CCEO trains its parameters, and hybridization of attention-guided deep broad convolutional neural network (ADBNet) [30] and deep channel-attention correlative capsule network (DCACorrCapsNet) [31] with Taylor series [32] develops DCABNet. In addition, CCEO is devised by merging the chronological concept with the CEO [33]. At the server, the average method performs local updation and aggregation. Schematic depiction of CCEO-DCABNet-enabled multiclass disease classification using FL is shown in Figure 2.

### 3.2. Training of Local Model by Local Data

The introduced CCEO-DCABNet was then trained via Federated Learning (FL), which comprises of four clients and one server. These clients represent independent hospitals, each with their own independent CXR datasets. The non-IID distribution method was used for data distribution, with disease examples distributed among clients differently to replicate practical medical settings. The global network was first shared with all clients, who trained on the local dataset for 5 epochs with a learning rate of 0.001 and a batch size of 64. Afterwards, only the model parameters were transferred back to the server after client-side training, ensuring that the patient’s raw information is not shared with anyone else. This training process was repeated for 50 communication rounds.

#### 3.2.1. Training of Every Node

Local node training on all devices is done at fixed intervals, and initialization is done by each node. Furthermore, overhead communication is minimized, and data secrecy is improved using collaborative training.

#### 3.2.2. Training Model

Classification of multiclass disease is performed in a training model, where input acquisition, pre-preparation, segmentation of the lung lobe, extraction of features, and disease classification are performed.

#### 3.2.3. Acquisition of Data for Intrusion Detection

Input data are gathered from the NIH Chest X-ray [24] and Detection and Segmentation of Radiographic Features of Pulmonary Edema dataset [25]. NIH Chest X-ray dataset [24] comprises 112,120 frontal images from 30,805 individuals, and the images are categorized as 15 labels. Metadata, like gender, patient age, image spacing, and view position, are accessible in csv type. Here, C specifies this dataset, whole images are denoted by ω, and $C_{\mu }$ implies $\mu^{m}$ image.

(2) $$C\;=\;\{C_{1},C_{2},\ldots ,C_{\mu },\ldots ,C_{\omega }\}$$

The Pulmonary Edema dataset [25] contains 1000 chest X-ray images from 741 individuals and was obtained from Version 2 of the Mendeley Data repository, with images extracted from the MIMIC database. It includes 4293 feature remarks, and every case depicts edema severity stage, no edema, interstitial edema, alveolar edema, or vascular congestion. The following expression represents dataset D.

(3) $$D\;=\;\{D_{1},D_{2},\ldots ,D_{\mu },\ldots ,D_{\xi }\}$$

It includes ξ images, and μ image is represented by $D_{\mu }$.

Here, images from the NIH Chest X-ray dataset [26] are used for disease classification. Moreover, similar processes are carried out by considering Pulmonary Edema dataset [25].

To mitigate data leakage risks from the federated learning nodes, it is strictly ensured that all data partitioning is done at the patient level, not the image level. As a result, several longitudinal or cross-sectional X-ray projections of the same unique patient identifier will always be placed in the training, validation, or test datasets. The split ratio used for both datasets is global and was applied at 70% training, 10% validation, and 20% independent test sets. In an attempt to simulate the Federated Learning environment in the clinical setting, the images were allocated across four different localized edge nodes in a Non-IID manner. The medical category and node allocation distribution are illustrated in Table 1.

#### 3.2.4. Input Image Pre-Preparation:

Pre-preparation improves quality of images by minimizing noise and artifacts. Here, denoising of the image is performed by a Gaussian filter [26] and sharpening is performed using multiscale unsharp masking [27].

1. Denoising using Gaussian Filter:
  The image $C_{\mu }$ is denoised using Gaussian filtering, where a Gaussian function is applied to suppress noise and smooth the image. This filtering operation produces a visually uniform blur, similar to the bokeh effect caused by object shadows under illumination. Consequently, the smoothing process enhances the perceptual quality of the image at multiple scales. The resulting denoised image is represented as $E_{\mu }$.
2. Sharpening using Multiscale Unsharp Masking:
  Multiscale unsharp masking is employed to enhance image sharpness by subtracting the blurred components from the original image. In this approach, three Gaussian kernels of different sizes are utilized to generate multiple blurred versions of the image. These multiscale blurred representations help preserve fine details while improving edge clarity and overall image contrast.

(4) $$E_{\mu \_Blur_{\varphi }}\left(\vartheta ,\rho \right)\;=\;Gau_{\varphi }\left(\vartheta ,\rho \right)*E_{\mu }\left(\vartheta ,\rho \right)$$

where $Gau_{\varphi }\left(\vartheta ,\rho \right)$ implies a Gaussian kernel function, count of filtering scale is denoted by φ, and $E_{\mu \_Blur_{\varphi }}\left(\vartheta ,\rho \right)$ specifies the blurred image. Three blurred images are computed, and are incorporated into a single image.

(5) $$E_{\mu \_Blur}\;=\;\sum_{\varphi =1}^{\Phi }E_{\mu \_Blur_{\varphi }}/\Phi$$

Subtraction of the blurred image from the input offers a sharpened image, where linear normalization is employed to uniformly distribute a set of pixels. Furthermore, sharpened images are obtained by

(6) $$F_{\mu }\;=\;E_{\mu }+\zeta \left\{E_{\mu }-E_{\mu \_Blur}\right\}*\upsilon$$

where ζ specifies linear normalization and υ represents a parameter to obtain a sharpened image $F_{\mu }$.

#### 3.2.5. Segmentation of Lung Lobe by MMPU-Net

Segmentation of lung lobes is needed to enhance the classification accuracy by precisely localizing abnormalities. Here, a sharpened image $F_{\mu }$ is fed to the MMPU-Net to segment lung lobe.

**Figure 3:**
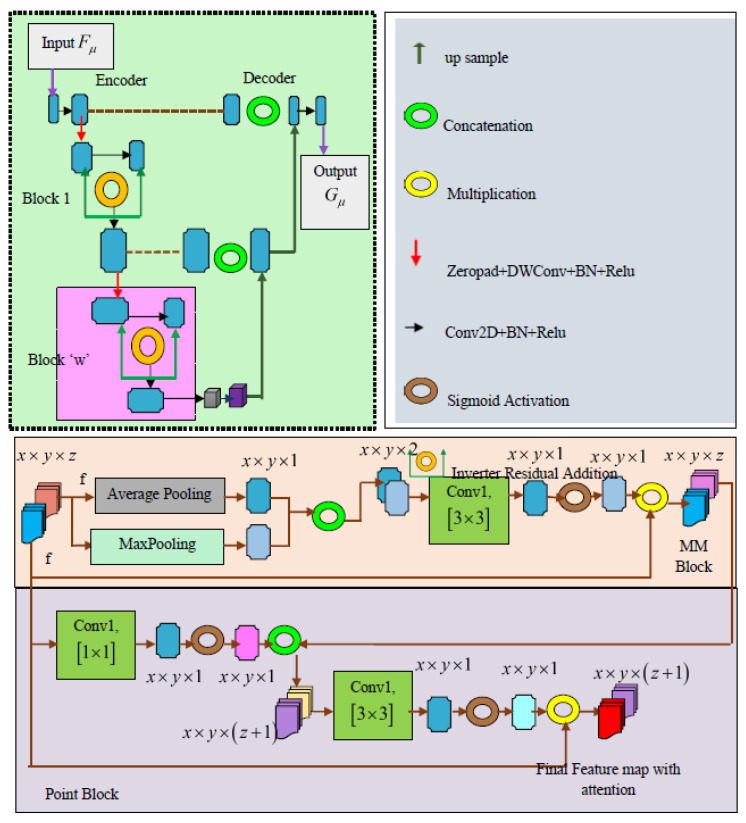
Structure of MMPU-Net.

1. Structure of MMPU-Net
  Sharpened image $F_{\mu }$ is subjected to MMPU-Net for segmenting lung lobe, and it is displayed in Figure 3. Coarse and fine segmentation are the two stages, in which fine segmentation is done by creating a predictive mask and then localizing the pancreas. After cropping the region of interest (ROI), bounding box of the pancreas is extracted to detect extreme coordinates. Finally, the cropped area is refined at a fine stage for obtaining the segmented output $G_{\mu }$. Encoder, inverted residual addition, decoder, and bottleneck are major parts of MMPU-Net. Encoder covers four stages, where blocks 0 to 13 are selected among the 17 blocks. Channel count of encoder layer is 32, and extracts transitional feature maps from four expanded ReLU blocks 13, 6, 3, and 1. Here, depthwise convolution (Conv) with stride 2 is employed for downsampling, and feature maps of such blocks are concatenated to feature map of upsampling. MMPU-Net includes inverted residual addition for preserving data and improving training stability. After yielding an intermediate outcome from Conv, the dimension adjustment performs direct addition between the input and intermediate output. Decoder of MMPU-Net is based on the U-Net structure, which includes skip contacts and progressive upsampling for restoring spatial details. The Conv filters of the decoder include 3 × 3 dimension kernel to offer even feature extraction. Moreover, the decoder performs gradual drop of feature channels by increasing spatial resolution. Point-block and MM-block are employed as an attention unit of the bottleneck unit. MM-block is used as a Conv-based attention with pooling paths, where the primary pathway depends on average pooling and secondary utilizes maxpooling. Here, input feature of the MM-block is represented by $f\in R^{x\times y\times z}$ and *f* undergoes average and max pooling. The outcome of these functions is merged to improve learned description features. Further, mean, and average pooling on the channel axis is computed by
  (7) $$f_{Mean}\;=\;\frac{1}{\Upsilon }\sum_{ℑ=1}^{\Upsilon }F_{\mu }\left(\tau_{1},\tau_{2},z\right)$$
  (8) $$f_{Max}\;=\;\max_{ℑ=1}^{\Upsilon }F_{\mu }\left(\tau_{1},\tau_{2},z\right)$$
  (9) $$f_{Fusion}\left(\tau_{1},\tau_{2}\right)\;=\;\left[\begin{array}{c} f_{Mean}\left(\tau_{1},\tau_{2}\right)\\ f_{Max}\left(\tau_{1},\tau_{2}\right) \end{array}\right]$$
  Here, z indicates channel $\left(\tau_{1},\tau_{2}\right)$ and specifies spatial indices of the features. Afterward, $f_{Fusion}$ is convolved to a single 3 × 3 kernel for adjusting its sizes and creating $K\in {ℜ}^{x\times y\times 1}$. Hence, output dimension of $f_{Fusion}$ is equal to dimensionality of K. The Conv function is given by
  (10) $$K_{Same}\left(\tau_{1},\tau_{2}\right)\;=\;\sum_{{℘}_{1}=-1}^{1}\sum_{{℘}_{2}=-1}^{1}Conv_{Ker}\left({℘}_{1},{℘}_{2}\right)\cdot F_{\mu }\left(\tau_{1}+{℘}_{1},\tau_{2}+{℘}_{2}\right)$$
  Here, $Conv_{Ker}\left({℘}_{1},{℘}_{2}\right)$ implies Conv kernel at $\left({℘}_{1},{℘}_{2}\right)$ and $F_{\mu }\left(\tau_{1}+{℘}_{1},\tau_{2}+{℘}_{2}\right)$. Using mean-max attention map $MM\in {ℜ}^{x\times y\times 1}$, sigmoid activation becomes
  (11) $$MM_{att}\;=\;af\left(K_{Conv}^{1\times 1}\right)$$

#### 3.2.6. Radiomic Feature Extraction from Segmented Image

Extraction of radiomic features [29] converts the images into high-dimensional geometrical features. Here, radiomic features are extracted from the segmented image $G_{\mu }$.

1. Compactness
  Compactness [29] is employed for measuring degree of curvature, and it is represented as the proportion of inner region of the image to the perimeter.
  (12) $$H_{1}\;=\;\frac{4\pi \cdot L(G_{\mu })}{pe{\left(G_{\mu }\right)}^{2}}$$
  Compactness is specified by $H_{1}$, implies area of $L(G_{\mu })$, and denotes the perimeter $pe(G_{\mu })$.
2. Solidity
  The fraction of inner region of segmented image to convex area is termed as solidity [29], and it is given by
  (13) $$H_{2}\;=\;\frac{L(G_{\mu })}{L_{Con}\left(G_{\mu }\right)}$$
  Solidity is denoted by $H_{2}$, and $L_{Con}\left(G_{\mu }\right)$ implies convex area of $G_{\mu }$.
3. Eccentricity
  Eccentricity [29] indicates $M_{1}$ a relation between the extensive to the shortest $M_{2}$ straight line in the image, as represented by
  (14) $$H_{3}\;=\;\sqrt{1-\frac{M_{1}{\left(G_{\mu }\right)}^{2}}{M_{2}{\left(G_{\mu }\right)}^{2}}}\left(M_{2}\left(G_{\mu }\right)\geq M_{1}\left(G_{\mu }\right)\right)$$
  Here, $H_{3}$ implies Eccentricity.
4. Roundness
  Roundness [29] is equivalent to compactness, but it utilizes perimeter of a convex instead of the region’s perimeter.
  (15) $$H_{4}\;=\;\frac{4\pi \cdot L(G_{\mu })}{pe_{Con}{\left(G_{\mu }\right)}^{2}}$$
  where $pe_{Con}$ symbolizes perimeter of a convex, $H_{4}$ and specifies Roundness.

Furthermore, $H_{\mu }$ radiomic feature vector is given by

(16) $$H_{\mu }\;=\;\left\{H_{1},H_{2},H_{3},H_{4}\right\}$$

#### 3.2.7. Classification of Multiclass Disease by CCEO-DCABNet

Classification of multiclass diseases is needed to categorize different disease types. Here, DCABNet performs multiclass disease classification, and it is trained by EECO.

##### Structure of DCABNet

The radiomic feature vector $H_{\mu }$ (which has a dimension of 1×4) is processed in parallel alongside the deep feature representations, rather than being directly concatenated to raw spatial image tensors.

The radiomic feature vector $H_{\mu }\;=\;\left\{H_{1},H_{2},H_{3},H_{4}\right\}$ represents a 1×4 numerical array containing global geometric properties (Solidity, Compactness, Eccentricity, and Roundness). To combine this statistical vector with the deep spatial abstractions generated within the network, $H_{\mu }$ is fed into two parallel pipelines:

- In DCACorrCapsNet: The 1×4 vector passes through a localized dense (fully connected) layer to project it into a higher-dimensional embedding space matching the capsule dimensions before undergoing spatial routing. The primary output $c_{1}$ represents a high-level capsule tensor.
- In ADBNet: The vector acts as a calibration weight via element-wise multiplication with the attention-guided convolutional maps ($g_{1}$ and $g_{2}$), acting as a structural regularizer.

Consequently, the inputs to the Taylor concept block are two distinct data types: $I_{1}$ (a regularized capsule feature tensor) and $I_{2}$ (a concatenated deep spatial-attention map).

**Figure 4:**
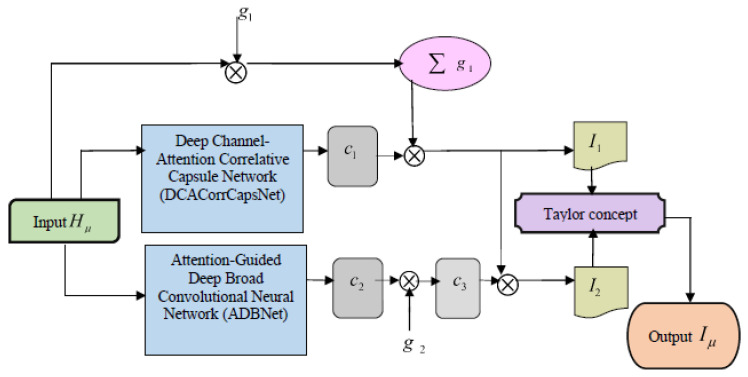
Description of the image.

The merging of DCACorrCapsNet [31] and ADBNet [30] using Taylor Series [32] develops DCABNet. Here, feature vector $H_{\mu }$ is fed to DCACorrCapsNet to obtain $c_{1}$. Moreover, $H_{\mu }$ is multiplied to $g_{1}$ (weight of DCACorrCapsNet) to create $\Sigma g_{1}$. Afterward, $\Sigma g_{1}$ and $c_{1}$ are multiplied to generate $I_{1}$. Likewise, $H_{\mu }$ is fed to ADBNet for attaining $c_{2}$, and then $c_{2}$ is multiplied to $g_{2}$ (weight of ADBNet) to attain $c_{3}$. Further, $c_{3}$ is multiplied to $I_{1}$ to attain $I_{2}$. Finally, $I_{1}$ and $I_{2}$ are fed to the Taylor series to achieve $H_{\mu }$. Figure 4 illustrates pictorial view of DCABNet.

1. DCACorrCapsNet
  DCACorrCapsNet [31] covers multi-feature extraction, hierarchical capsule, and classification modules, where the feature vector $H_{\mu }$ is considered as input. Multi-feature extraction performs augmentation, channel attention, and the Fisher vector module. Augmentation minimizes overfitting problems and uses geometric transformations to enhance the image. In Fisher vector, $L_{2}$ regularization normalizes numerical distribution. Conv kernels in the channel attention stage generate feature maps by aggregation of spatial dimensionality. The initial capsule constructs a set of parallel Conv layers, where each capsule in the correlative capsule layer independently extracts salient attributes and local structural details. In the classification module, outcome of the multi-level capsule with the feature extractor is applied to DeepGBM to obtain $I_{1}$.
  (17) $$I_{1}\;=\;\left(g_{1}^{T}\times J\left(N\left[H_{\mu },\varphi^{Gp}\right]\right)+g_{1_{0}}\right)\times \left(\Sigma g_{1}\times H_{\mu }\right)$$
  Here, weight is portrayed by $g_{1}$.
2. ADBNet
  ADBNet [30] improves feature depiction by including a deep network with attention functions. Input $H_{\mu }$ is fed to ADBNet to attain $I_{2}$, where the residual module attains superior learning from prior layers. It also contains different processing layers for refining input. In attention guided phase, CBAM focuses on informative details to suppress less appropriate features. Integration of modified CBAM with CNN is employed for learning rich descriptions of salient features. Deep, broad stage offers the learning of broader spectrum of data using a deep network style. Here, input features are fed to sequence of Conv and activation layers to create intermediate feature maps. Merging of fully connected and combination of global average layer creates a feature *e*.
  (18) e=FC(h(i))
  Here, FC implies a fully connected layer, as well as output, input, and intermediate features are denoted by *e*, *i* and *h*. The outcome of ADBNet $I_{2}$ is given by.
  (19) $$I_{2}\;=\;conc\left(H_{\mu },e\right)*g_{2}*I_{1}$$
  (20) $$I_{2}\;=\;conc\left(H_{\mu },e\right)*g_{2}*\left(g_{1}^{T}\times J\left(N\left[H_{\mu },\varphi^{Gp}\right]\right)+g_{1_{0}}\right)\times \left(\Sigma g_{1}\times H_{\mu }\right)$$
  Here, conc specifies the concatenation function.
3. Taylor concept
  Taylor concept [32] specifies a mathematical illustration of an infinite series, where $I_{1}$ and $I_{2}$ are applied to the Taylor series to achieve the detected output $I_{\mu }$.
  Mathematically, the hybrid feature map $I_{\mu }$ is derived by approximating the underlying non-linear interaction function via a second-order Taylor series expansion centered around a localized deep feature consensus λ:
  (21) $$I_{\mu }\approx f\left(\lambda \right)+f^{\prime }\left(\lambda \right)\left(I_{1}-\lambda \right)+\frac{1}{2!}f^{\prime \prime }\left(\lambda \right){\left(I_{2}-\lambda \right)}^{2}$$
  where $I_{1}$ and $I_{2}$ represent the multi-feature capsule routing map from the DCACorrCapsNet and the attention-guided map from the ADBNet, respectively. By projecting these variables into an interrelated Taylor expansion space, the network preserves structural gradients and bridges the optimization void between mathematical radiomics and neural attention. This unified optimization loop forms the core structural innovation of the CCEO-DCABNet framework, preventing cross-module information loss during distributed federated rounds
  (22) $$\begin{array}{c} I_{\mu }\;=\;2\left(g_{1}^{T}\times J\left(N\left[H_{\mu },\varphi^{Gp}\right]\right)+g_{1_{0}}\right)\times \left(\Sigma g_{1}\times H_{\mu }\right)-\;\;\;\\ \;\;\;\;\;\left(conc\left(H_{\mu },e\right)*g_{2}*\left(g_{1}^{T}\times J\left(N\left[H_{\mu },\varphi^{Gp}\right]\right)+g_{1_{0}}\right)\times \left(\Sigma g_{1}\times H_{\mu }\right)\right) \end{array}$$
  From Equation (22), outcome of DCABNet is obtained.

##### Training of DCABNet Using CCEO

CCEO trains DCABNet for achieving optimal results in disease classification. The chronological idea is merged with the CEO [31] to develop CCEO, where chaotic dynamics are the basis of the CEO. Chaotic evolution of a two-dimensional distinct memristive map is an inspiration to develop CEO. Using hyperchaotic features of the memristive map, modelling of the CEO offers random search instructions in evolutionary procedures. The integration of mutation and crossover from differential evolution (DE) develops CEO. Furthermore, a hyperchaotic map creates a relation between individuals and integrates memory function for improving search diversity and exploring its population to promising search regions. Every member of the CEO creates various directions of chaotic evolution for increasing the exploitation and exploration capabilities of present members. Moreover, the demonstrates better robustness and global searching capacity. In addition, merging of the chronological concept to ECO lessens chaotic oscillations and further improves consistency and faster performance. Steps followed in CCEO are described below.

Step 1: Initialization

Initially, the CEO comprises basic constraints and candidate solutions to control the search process. Further, population members are initiated as

(23) $$P\;=\;\{P_{1},P_{2},\ldots ,P_{m},\ldots ,P_{\upsilon }\}$$

Here, the overall population is specified by υ and $p_{m}$ implies the present population.

(24) $$P_{v}\;=\;§+\Psi *\left(\lambda -§\right)$$

Random value is represented by Ψ and upper and lower bounds are denoted by λ and §.

Step 2: Fitness Function

Ideal solution of DCABNet is attained using the fitness function, where mean square error (MSE) of the output from DCABNet is used to compute the fitness function.

(25) $$Fitness\;=\;\frac{1}{\sigma }\sum_{\mu =1}^{\sigma }{\left(I_{\mu }^{*}-I_{\mu }\right)}^{2}$$

where $I_{\mu }$ specifies an outcome of DCABNet, and the expected output is denoted by $\left(I_{\mu }^{*}\right)$.

Step 3: Mutation Operation

Mutation of the CEO utilizes a unified search region, and muted individuals and present individuals are given by

(26) $$Q_{j+1}\;=\;Q_{j}+q_{st}\cdot l_{j}$$

Here, muted individuals are denoted by $Q_{j+1}$ and $Q_{j}$ specifies present individuals. Moreover, dimension of the search step is indicated by $q_{st}$, and direction of evolution is portrayed by *l*.

(27) $$\left\{\begin{array}{c} Q_{j}^{\prime }\;=\;\frac{Q_{j}-V}{U-V}-0.5\\ R_{j}^{\prime }\;=\;\frac{R_{j}-V}{U-V}\times 0.5-0.25 \end{array}\right.$$

Here, $Q_{j}^{\prime }$ and $R_{j}^{\prime }$ implies chaotic original locations after mapping. The terms U and V specifies upper and lower limits. Furthermore, a count of samples is presented, where $Q\_Chaos\;=\;\{Q\_{Chaos}^{1},\ldots ,Q\_{Chaos}^{O}\}$ and $R\_Chaos\;=\;\{R\_{Chaos}^{1},\ldots ,R\_{Chaos}^{O}\}$ are created. Afterward, these individuals are mapped to the original value $Q\_{Chaos}^{\prime }=\left\{Q\_{Chaos}^{1^{\prime }},\ldots ,Q\_{Chaos}^{O^{\prime }}\right\}$ and $R\_{Chaos}^{\prime }=\left\{R\_{Chaos}^{1^{\prime }},\ldots ,R\_{Chaos}^{O^{\prime }}\right\}$ and $R\_{Chaos}^{\prime }=\left\{R\_{Chaos}^{1^{\prime }},\ldots ,R\_{Chaos}^{O^{\prime }}\right\}$ and by inverse mapping.

(28) $$\left\{\begin{array}{c} Q\_{Chaos}^{O^{\prime }}\;=\;\left(Q\_{Chaos}^{O}+0.5\right)\times \left(U-V\right)+V\\ R\_{Chaos}^{O^{\prime }}\;=\;\left(R\_{Chaos}^{O}+0.25\right)\times 2\times \left(U-V\right)+V \end{array}\right.$$

where $Q\_{Chaos}^{O^{\prime }}$ and $R\_{Chaos}^{O^{\prime }}$ create evolutionary direction of $Q_{j}$ and $R_{j}$.

(29) $$\left\{\begin{array}{c} l_{Q,j}^{O}\;=\;Q\_{Chaos}^{O^{\prime }}-Q_{j}\\ l_{R,j}^{O}\;=\;R\_{Chaos}^{O^{\prime }}-R_{j} \end{array}\right.$$

Here, o={1,2,…,O} and evolutionary direction of $Q_{j}$ and $r_{j}$ are denoted by $l_{Q,j}^{o}$ and $l_{R,j}^{o}$. Hence, mutation of the CEO becomes

(30) $$\left\{\begin{array}{c} Q_{j+1}^{o}\;=\;Q_{j}+q_{st}\cdot \left(Q\_{Chaos}^{o^{\prime }}-Q_{j}\right)\\ R_{j+1}^{o}\;=\;R_{j}+q_{st}\cdot \left(R\_{Chaos}^{o^{\prime }}-R_{j}\right) \end{array}\right.$$

Dimension of 0 mutant members lies between [0, 1] interval. Consider $Q_{j}$, updated position becomes

(31) $$Q_{j+1}^{o}\;=\;Q_{j}+q_{st}\cdot \left(Q\_{Chaos}^{o^{\prime }}-Q_{j}\right)$$

Let, $Q_{j+1}^{o}$=$Q_{j+1}$, Equation (31) becomes

(32) $$Q_{j+1}\;=\;Q_{j}+q_{St}\cdot Q\_{Chaos}^{o}-k\cdot Q_{j}$$

(33) $$Q_{j+1}\;=\;Q_{j}\left[1-q_{St}\right]+q_{St}\cdot Q\_{Chaos}^{o}$$

Applying the chronological concept

(34) $$Q_{j+1}\;=\;\frac{Q_{j+1}+Q_{j+1}}{2}$$

During *J*-th iteration,

(35) $$Q_{j}\;=\;Q_{j-1}\left[1-q_{St}\right]+q_{St}\cdot Q\_{Chaos}^{o}$$

Substitute Equation (35) in Equation (33)

(36) $$Q_{j+1}\;=\;\left(Q_{j-1}\left[1-q_{St}\right]+q_{St}\cdot Q\_{Chaos}^{o^{\prime }}\right)\left[1-q_{St}\right]+q_{St}\cdot Q\_{Chaos}^{o^{\prime }}$$

(37) $$Q_{j+1}\;=\;Q_{j-1}\cdot {\left[1-q_{St}\right]}^{2}+q_{St}\cdot Q\_{Chaos}^{o^{\prime }}\cdot \left[1-q_{St}\right]+q_{St}\cdot Q\_{Chaos}^{o^{\prime }}$$

Substitute Equations (33) and (37) to Equation (34)

(38) $$Q_{j+1}\;=\;\frac{1}{2}\left[\begin{array}{c} (Q_{j}\left[1-q_{St}\right]+q_{St}\cdot Q\_{Chaos}^{o^{\prime }}+Q_{j+1})+\\ Q_{j-1}{\left[1-q_{St}\right]}^{2}+q_{St}\left[1-q_{St}\right]\cdot Q\_Chaos^{o^{\prime }}+q_{St}\cdot Q\_{Chaos}^{o^{\prime }} \end{array}\right]$$

(39) $$Q_{j+1}\;=\;\frac{1}{2}\left[\begin{array}{c} \left(Q_{j}\left[1-q_{St}\right]+2q_{St}\cdot Q\_{Chaos}^{o^{\prime }}+Q_{j+1}\right)+\\ Q_{j-1}{\left[1-q_{St}\right]}^{2}+q_{St}\left[1-q_{St}\right]\cdot Q\_{Chaos}^{o^{\prime }} \end{array}\right]$$

Updated solution of CCEO is given in Equation (39). To improve local development, the best solution fo the present population becomes

(40) $$\left\{\begin{array}{c} {\hat{Q}}_{j+1}^{o}\;=\;{Best}_{j}+q_{St}\cdot \left(Q\_{Chaos}^{o^{\prime }}-Q_{j}\right)\\ {\hat{R}}_{j+1}^{o}\;=\;{Best}_{j}+q_{St}\cdot \left(R\_{Chaos}^{o^{\prime }}-R_{j}\right) \end{array}\right.$$

Best solution for the present population is specified by $Best_{j}$.

Step 4: Crossover Operation

Binomial crossover of $\left(Q_{j},⊗Q_{j+1}^{o}\right)$ and $\left(R_{j},⊗R_{j+1}^{o}\right)$ creates trial vector $Q\_{\mathrm{Trial}}_{j}^{o}\;=\;\left(Q\_{\mathrm{Trial}}_{1,j}^{o},Q\_{\mathrm{Trial}}_{2,j}^{o},\ldots ,Q\_{\mathrm{Trial}}_{Dim,j}^{o}\right)\;\mathrm{and}\;Q\_{\mathrm{Trial}}_{j}^{o}\;=\;(Q\_{\mathrm{Trial}}_{1,j}^{o},Q\_{\mathrm{Trial}}_{2,j}^{o},$$\ldots ,Q\_{\mathrm{Trial}}_{Dim,j}^{o})$. Consider $\left(Q_{j},⊗Q_{j+1}^{o}\right)$, crossover becomes

(41) $$\left\{\begin{array}{cc} Q\_{\mathrm{Trial}}_{m,j}^{o}, & \mathrm{If}\;\left({Rnd}_{m}\left(0,1\right)\leq S\right)\;\mathrm{or}\;\left(m\;=\;m_{Rnd}\right)\\ Q_{m,j}, & \mathrm{Otherwise} \end{array}\right.$$

Here, dimension of the optimization problem is denoted by Dim, $m\;=\;1,2,\ldots ,Dim;\;m_{Rnd}$ denotes an integer that is selected randomly at [1,Dim] interval. Random value $Rnd_{m}\left(0,1\right)$ is created for every member, and S specifies the crossover control factor set as [0, 1].

Step 5: Selection Operation

For individuals $Q_{j}$ and $R_{j}$, trial vectors $Q\_{\mathrm{Trial}}_{j}^{o}$ and $R\_{\mathrm{Trial}}_{j}^{o}$ are created. CEO utilized Greedy factor for selecting experimental vector, and the selection operation becomes

(42) $$Q_{j+1}\;=\;\left\{\begin{array}{cc} Q\_{\mathrm{Trial}}_{j}^{*}, & \mathrm{If}\;X\left(Q\_{\mathrm{Trial}}_{j}^{*}\right)\leq X\left(Q_{j}\right)\\ Q_{j}, & \mathrm{Otherwise} \end{array}\right.$$

(43) $$R_{j+1}\;=\;\left\{\begin{array}{cc} R\_{\mathrm{Trial}}_{j}^{*}, & \mathrm{If}\;X\left(R\_{\mathrm{Trial}}_{j}^{*}\right)\leq X\left(R_{j}\right)\\ R_{j}, & \mathrm{Otherwise} \end{array}\right.$$

Here, $Q\_{\mathrm{Trial}}_{j}^{*}$ and $R\_{\mathrm{Trial}}_{j}^{*}$ implies best trial vector $Q\_{\mathrm{Trial}}_{j}^{o}\;=\;\left(Q\_{\mathrm{Trial}}_{1,j}^{o},Q\_{\mathrm{Trial}}_{2,j}^{o},\ldots ,Q\_{\mathrm{Trial}}_{Dim,j}^{o}\right)$

Step 6: Re-estimation of fitness

Until getting an ideal solution, re-computation of fitness is repeated.

Step 7: Termination

CCEO reaches termination when executing highest level of iterations to obtain an optimal result. Algorithm 1 illustrates Pseudocode of CCEO.
**Algorithm 1:** Pseudocode for CCEO.1:**Input:** Count of chaotic samples $C_{c}$, population dimension, evolution function2:**Output:** Updated solution of CCEO $O_{j+1}$3:j←1                             ▹ initial iteration4:Initialize population and evaluate individuals5:$Evolution\leftarrow O_{0}$6:**while** Evaluation < Max_Evaluation **do**7:      **repeat**8:            Select two individuals $\left[Q_{j},R_{j}\right]$9:            Perform interval mapping $\left[Q_{j}^{\prime },R_{j}^{\prime }\right]$10:            Compute chaotic coordinates $C\left[Q\_\mathrm{Chaos},R\_\mathrm{Chaos}\right]$11:            Execute actual position update $\left[Q\_{\mathrm{Chaos}}^{\prime },R\_{\mathrm{Chaos}}^{\prime }\right]$12:            Corresponding optimization (evaluate fitness)13:            **if** Rnd < 0.5 **then**14:                Compute updated solution $O_{j+1}$ by Equation (37)15:            **else**16:                Execute mutation process $\left[Q_{j+1},R_{j+1}\right]$ by Equation (38)17:            **end if**18:            S←Rnd(0,1)19:            Execute crossover process $\left[Q_{{\mathrm{Trial}}_{j}}^{o},R_{{\mathrm{Trial}}_{j}}^{o}\right]$20:            Execute selection process to obtain $\left[Q_{j+1},R_{j+1}\right]$21:            Update the population (population, fitness)22:            Evolution←Evolution+2∗O23:      **until** every individual is selected once24:      j←j+125:**end while**26:**return **$O_{j+1}$

### 3.3. Aggregation on Global Server

**Figure 5:**
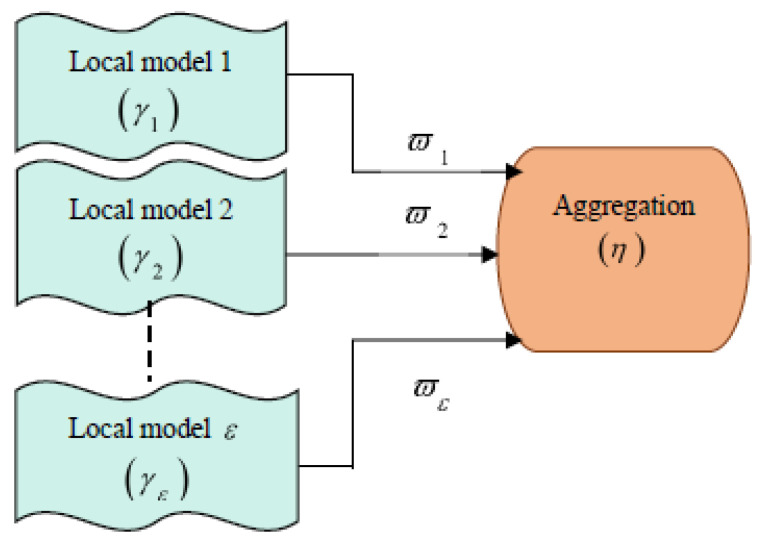
Graphical illustration for aggregation.

Aggregation on the server merges weights of all local models, and updates the global server. Here, $\omega_{1},\omega_{2},\ldots ,\omega_{\varepsilon }$ specifies an average weight model $\gamma_{1},\gamma_{2},\ldots ,\gamma_{\varepsilon }$. Graphical depiction of aggregation is given in Figure 5, and weight aggregation on the server becomes

(44) $$\eta \;=\;\sum_{\kappa =1}^{\lambda }\omega_{\kappa }*\gamma_{\kappa }$$

where η implies aggregation.

Local training is done on every device, and it is fed to the server to perform aggregation. Also, average weights are fed to update the local model for maintaining secrecy.

## 4. Results and Discussion

Result and discussion of CCEO-DCABNet-enabled multiclass disease classification are described. Furthermore, dataset details, implementation tool, and assessment of CCEO-DCABNet using K-Fold and federated assessment are illustrated.

The experiments were conducted using a global server equipped with an Intel Xeon CPU, 64 GB RAM, and an NVIDIA RTX 3090 GPU with 24 GB memory, running on Windows 11 operating system. The local server consisted of an Intel Core i7 processor, 16 GB RAM, and an NVIDIA RTX 3060 GPU. The implementation was carried out using Python 3.10 with the TensorFlow framework.

### 4.1. Experimental Setup

CCEO-DCABNet-enabled multiclass disease classification is implemented in PYTHON tool, and parameter details of CCEO and DCABNet are represented in Table 2.

### 4.2. Description of Dataset

Inputs are acquired from the NIH Chest X-ray [24] and Pulmonary Edema dataset [25].

#### 4.2.1. NIH Chest X-Ray Dataset

NIH Chest X-ray dataset [24] includes 112,120 frontal images from 30,805 individuals, and resolution of images is 1024 × 1024 pixels. Further, images are categorized into 15 labels, and these labels are extracted from corresponding radiology descriptions. Metadata, like gender, patient age, image spacing, and view position, are accessible in csv type. Moreover, a randomly sampled subgroup of 5606 images offers rapid experimentation.

#### 4.2.2. Pulmonary Edema Dataset

The dataset in [25] is designed to train and evaluate pulmonary edema and assess its severity level. It includes 1000 images from 741 individuals, which are found in the MIMIC database. Moreover, it comprises 4293 feature remarks, and every case depicts edema severity stage, no edema, interstitial edema, alveolar edema, or vascular congestion. Hence, it is a valuable database for segmenting features, classifying diseases, and grading edema severity.

### 4.3. Metrics for Evaluation

The effectiveness of CCEO-DCABNet is appraised by the following measures.

#### 4.3.1. Accuracy

Accuracy [34] is portrayed as the rate of exactly detected samples from the whole samples. Expression for accuracy is

(45) $$\mathrm{Accuracy}\;=\;\frac{\chi_{Pv}+\zeta_{Pv}}{\chi_{Pv}+\zeta_{Pv}+\chi_{Nv}+\zeta_{Nv}}$$

Here, $chi_{Pv}$ is True positive, $\zeta_{Pv}$ is false positive, $chi_{Nv}$ is true negative, and $\zeta_{Nv}$ is false negative.

#### 4.3.2. TPR

TPR [34] defines a percentage of count of precisely identified positives from the entire positive samples.

(46) $$TPR\;=\;\frac{\chi_{Pv}}{\chi_{Pv}+\zeta_{Nv}}$$

#### 4.3.3. TNR

Rate of precisely identified negative samples from all negative samples is represented as TNR [34], and it is specified as

(47) $$TNR\;=\;\frac{\chi_{Nv}}{\chi_{Nv}+\zeta_{Pv}}$$

### 4.4. Experimental Outcome

**Figure 6:**
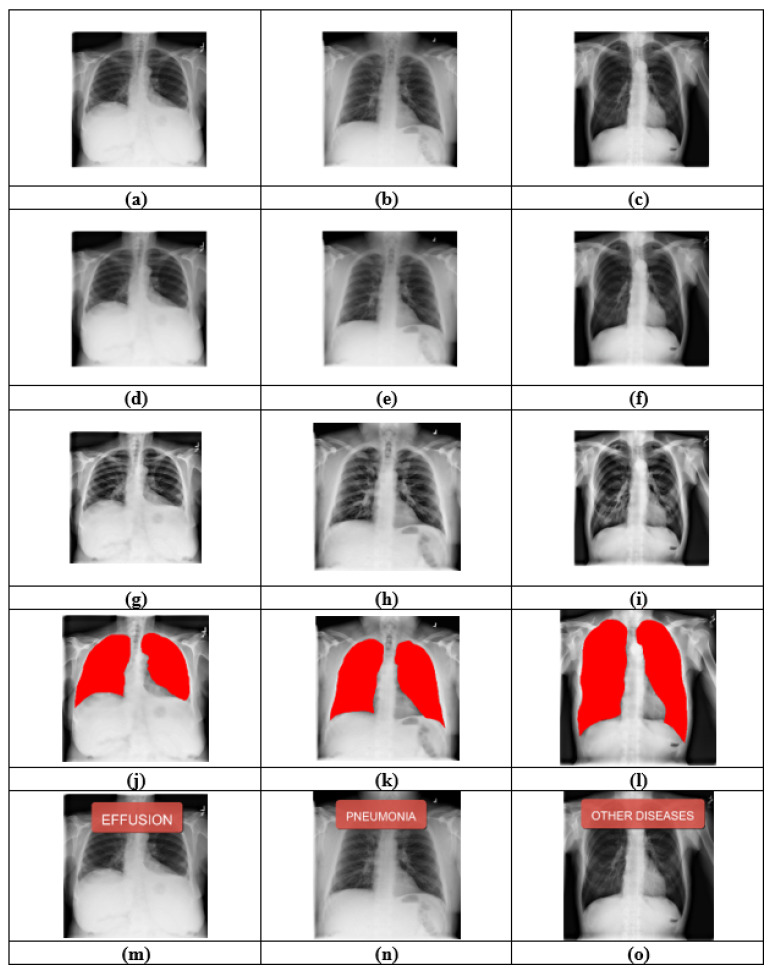
Experimental output of CCEO-DCABNet by NIH Chest X-ray dataset, (**a**) input-1, (**b**) input-2, (**c**) input-3, (**d**) denoised-1, (**e**) denoised-2, (**f**) denoised-3, (**g**) sharpened-1, (**h**) sharpened-2, (**i**) sharpened-3, (**j**) segmented-1, (**k**) segmented-2, (**l**) segmented-3, (**m**) detected output-1, (**n**) output-2, (**o**) output-3 images.

Figure 6 depicts experimental outcome of CCEO-DCABNet by NIH Chest X-ray Dataset. Figure 6a,d,g,j,m shows input, denoised, sharpened, segmented, and output image-1. Input, denoised, sharpened, segmented, and output image-2 are depicted in Figure 6b,e,h,k,n. Moreover, Figure 6c,f,i,l,o portray input, denoised, sharpened, segmented, and output image-3.

**Figure 7:**
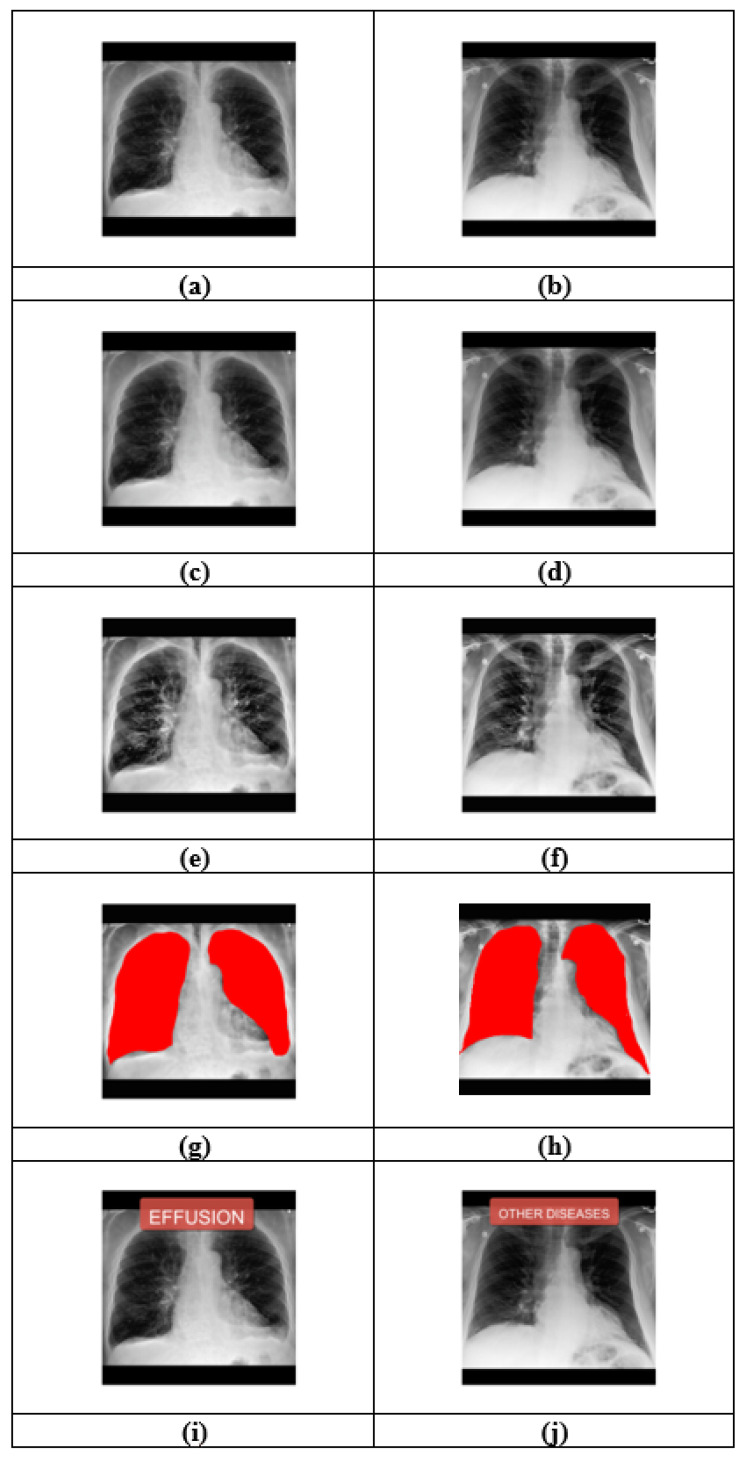
Experimental output of CCEO-DCABNet using Pulmonary Edema dataset, (**a**) input-1, (**b**) input-2, (**c**) denoised-1, (**d**) denoised-2, (**e**) sharpened-1, (**f**) sharpened-2, (**g**) segmented-1, (**h**) segmented-2, (**i**) output-1, (**j**) output-2 images.

Experimental result of CCEO-DCABNet using Pulmonary Edema dataset is shown in Figure 7. Input, denoised, sharpened, segmented, and output-1 images are illustrated in Figure 7a,c,e,g,i. Figure 7b,d,f,h,j display input, denoised, sharpened, segmented, and output-2 images.

### 4.5. Explainability Assessment

**Figure 8:**
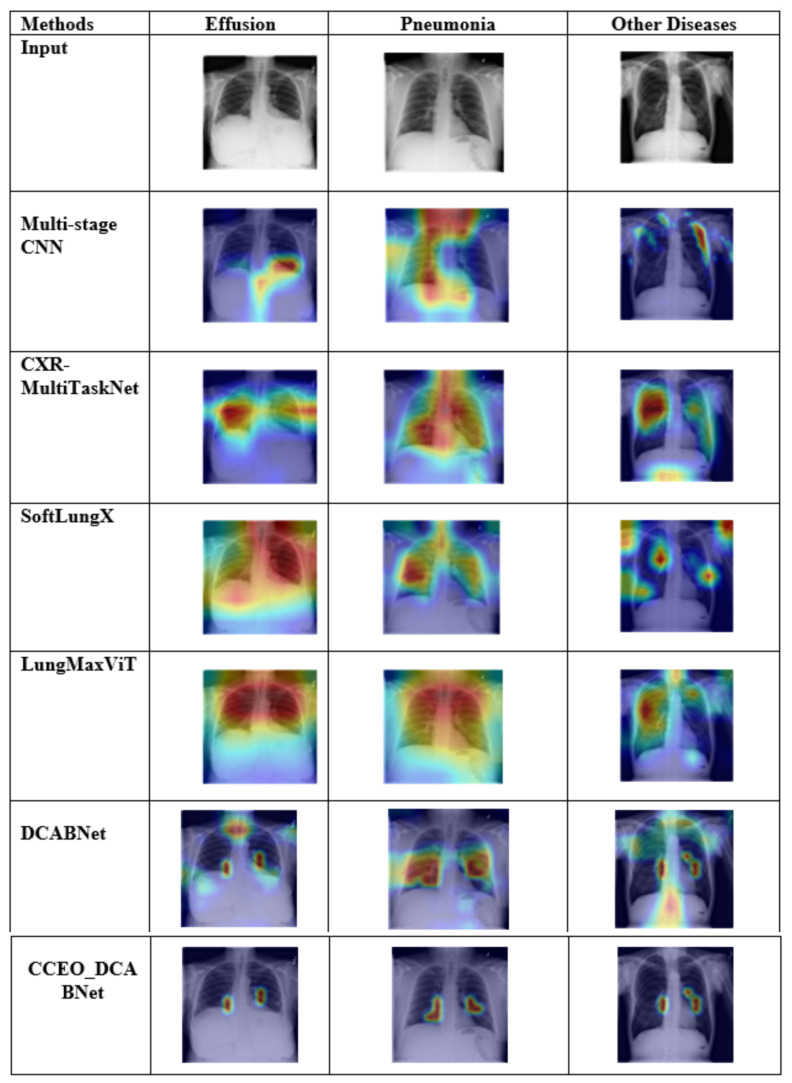
Grad cam visualization of CCEO-DCABNet using NIH Chest X-ray dataset.

**Figure 9:**
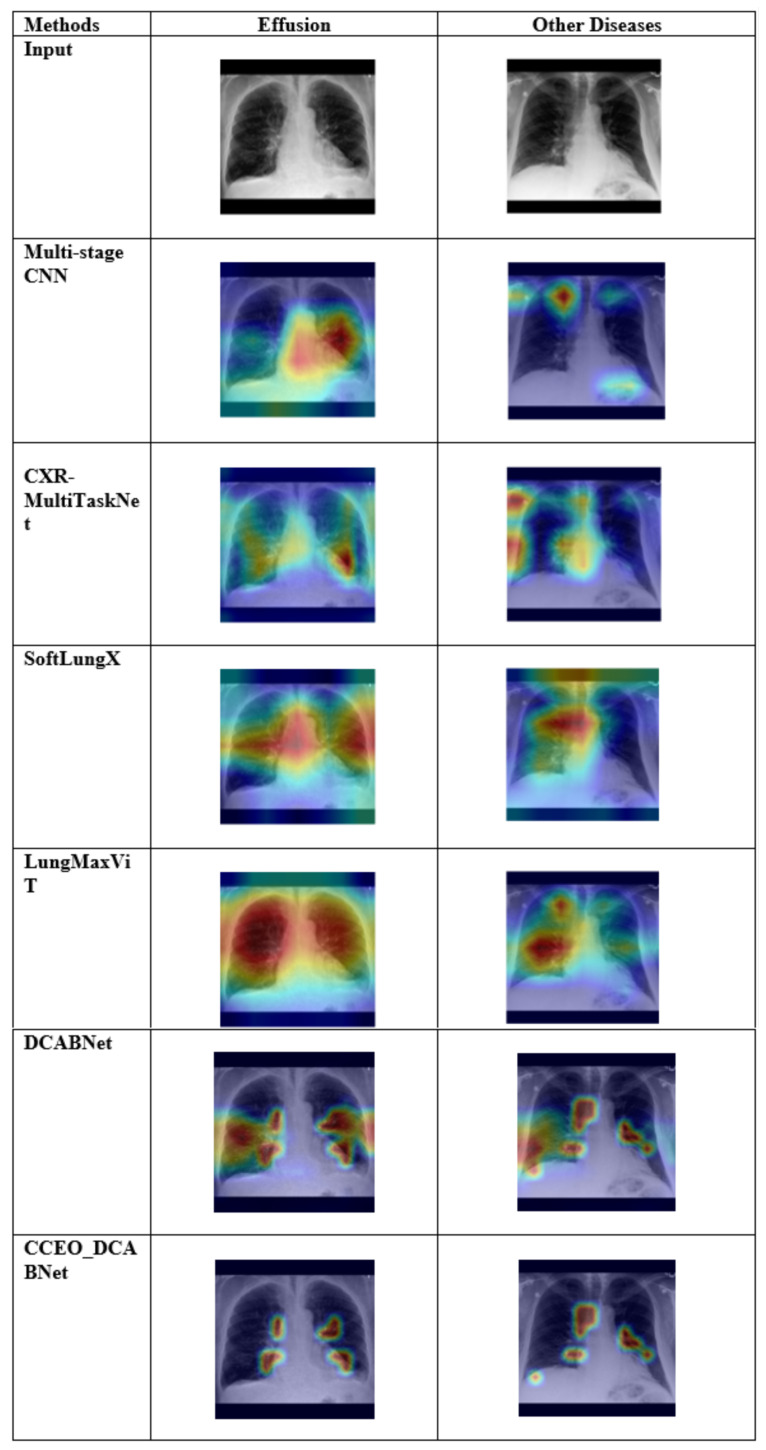
Grad cam visualization of CCEO-DCABNet using Pulmonary Edema dataset.

Detailed interpretation of feature contribution in CCEO-DCABNet is obtained by gradient-weighted class activation mapping (Grad-CAM). To identify the exact region in disease classification, Grad-CAM analyzes the model’s learning and finds exact features. Moreover, heatmap activation focuses on the affected area and provides an explanation of feature contribution. In medical assessment, pictorial descriptions relate medical authorities and AI to enable precise analysis. Also, Grad-CAM generates a heatmap overlapping and decision-making using highlighted parts. Moreover, images using the NIH Chest X-ray and Pulmonary Edema datasets are shown in Figure 8 and Figure 9.

### 4.6. ROC Curve

**Figure 10:**
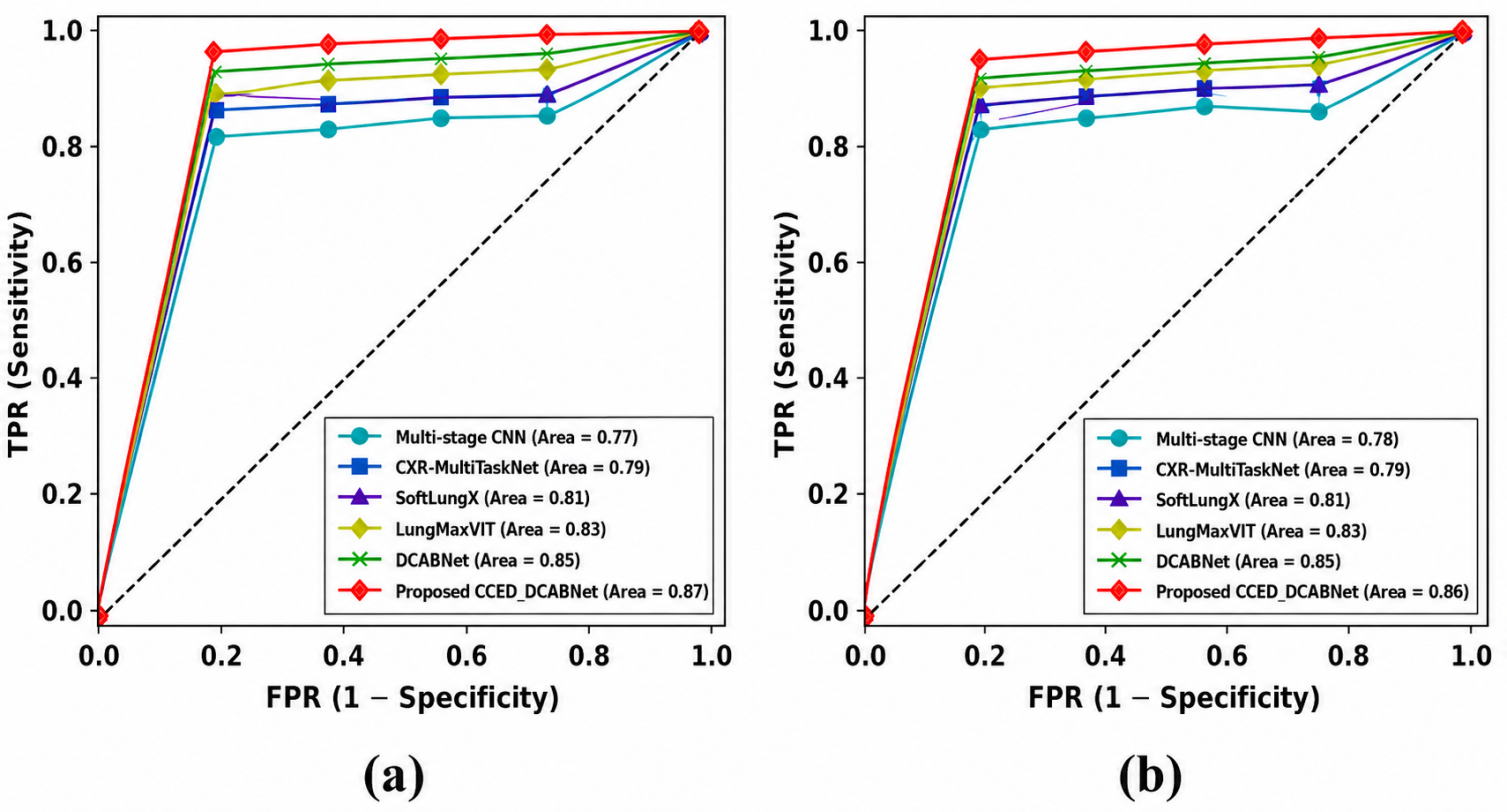
ROC Curve of CCEO-DCABNet using (**a**) NIH Chest X-ray dataset, (**b**) Pulmonary Edema dataset.

ROC curve of CCEO-DCABNet is portrayed in Figure 10, and it relates to false positive rate (FPR) and TPR. Here, the dashed line with AUC = 0.5 implies random classification of CCEO-DCABNet, and detected values above this line show discriminative capacity of the model. Figure 10a illustrates the ROC curve based on the NIH Chest X-ray dataset, where TPR attained by the existing and CCEO-DCABNet models are 1 when FPR = 1. Also, TPR of 0.978 is attained by CCEO-DCABNet, and values achieved by the multi-stage CNN is 0.863, CXR-MultiTaskNet is 0.876, SoftLungX is 0.902, LungMaxViT is 0.935, and DCABNet is 0.969 FPR = 0.8. Figure 10b depicts the ROC curve using the Pulmonary Edema dataset. For FPR = 1, CCEO-DCABNet and existing methods achieve the TPR of 1, as well as TPR of 0.853, 0.876, 0.904, 0.935, 0.961, and 0.978 are achieved by CCEO-DCABNet, and other models for FPR = 0.8.

### 4.7. Confusion Matrix

**Figure 11:**
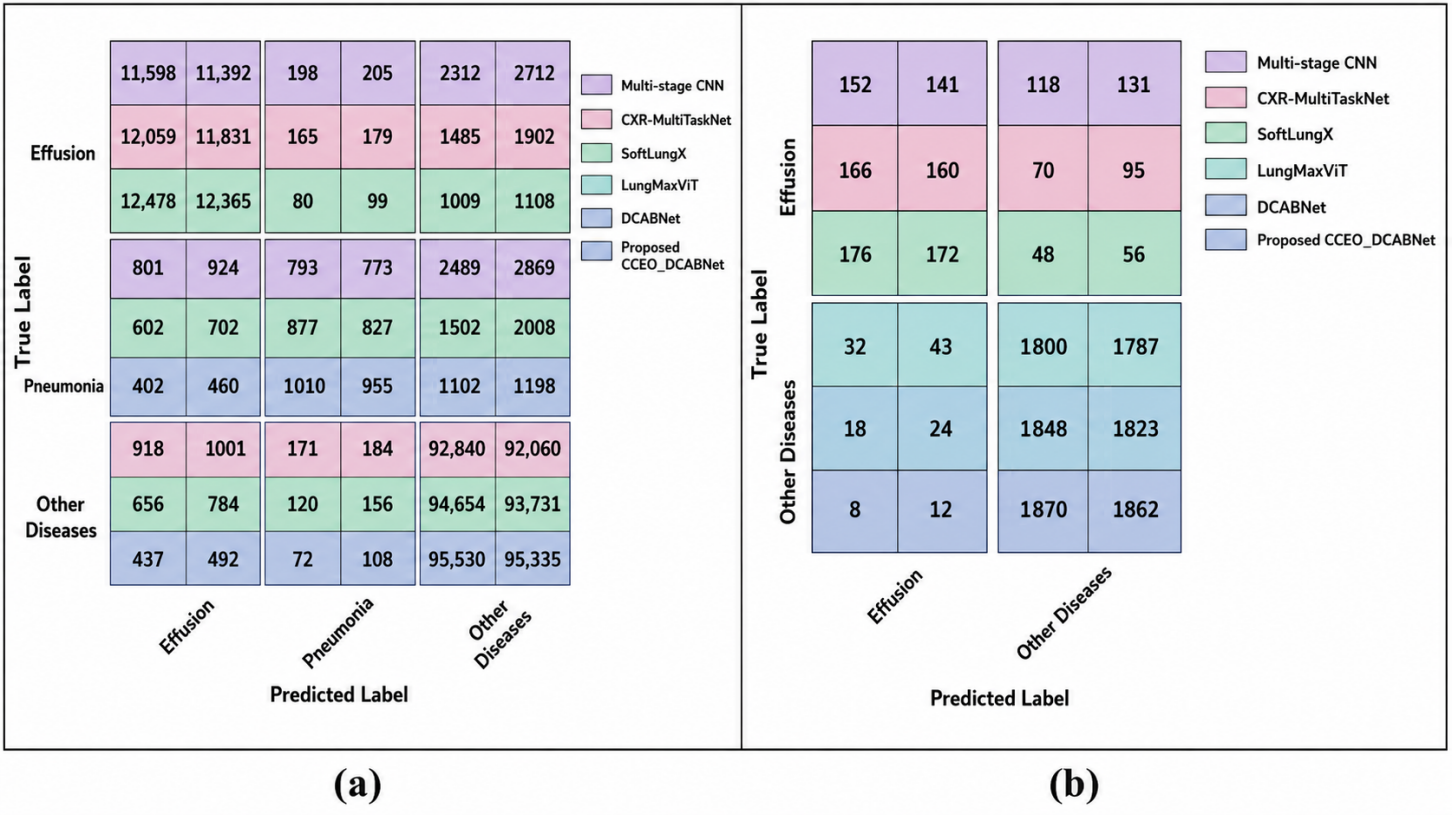
Confusion Matrix of CCEO-DCABNet using Edema dataset, (**a**) NIH Chest X-ray dataset, (**b**) Pulmonary Edema dataset.

Figure 11 deliberates confusion matrix of CCEO-DCABNet. Confusion matrix of CCEO-DCABNet using the NIH Chest X-ray dataset is portrayed in Figure 11a, which precisely classifies 71,993 samples as effusion, 5235 as pneumonia, and 564,150 as other diseases. Here, CCEO-DCABNet classifies 12,478 samples as effusion, and the remaining are classified by existing models. Among 5235 pneumonia samples, 1010 are precisely classified by CCEO-DCABNet. Moreover, CCEO-DCABNet classifies 95,530 samples as other diseases.

Confusion matrix using the Pulmonary Edema dataset is given in Figure 11b. This plot shows precise classification of Effusion and other diseases by CCEO-DCABNet and existing models. Here, 167 samples are precisely classified as Effusion, in which CCEO-DCABNet correctly identifies 176 samples. Moreover, 10,990 samples are clearly classified as other diseases, where 1870 are identified by CCEO-DCABNet.

### 4.8. Assessment of CCEO-DCABNet

Federated and K-Fold assessment of CCEO-DCABNet is described using different metrics.

#### 4.8.1. Federated Assessment

Federated assessment of CCEO-DCABNet is done by considering the NIH Chest X-ray and Pulmonary Edema datasets, where estimation is carried out by considering four chunks.

**Figure 12:**
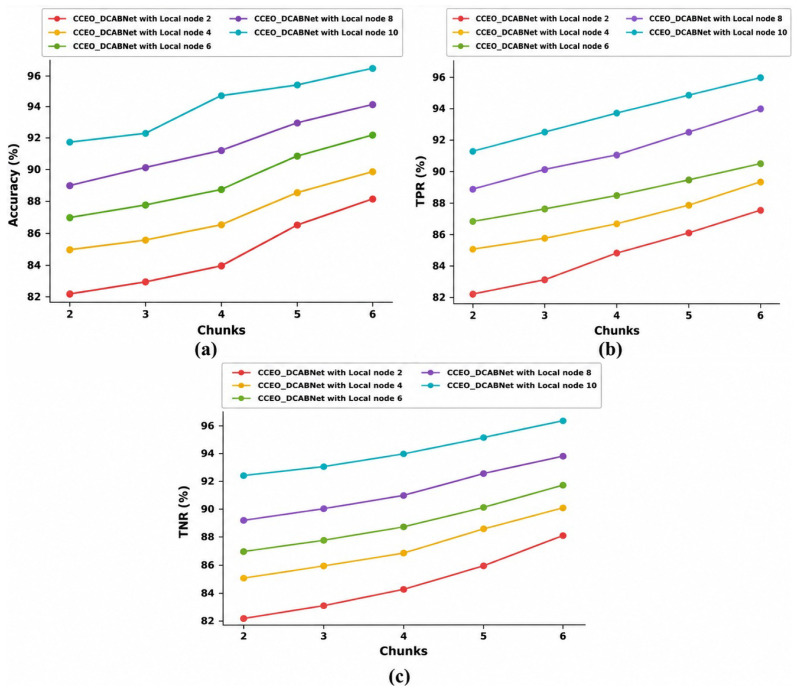
Federated Assessment of CCEO-DCABNet using NIH Chest X-ray dataset, (**a**) accuracy, (**b**) TPR, (**c**) TNR.

**Figure 13:**
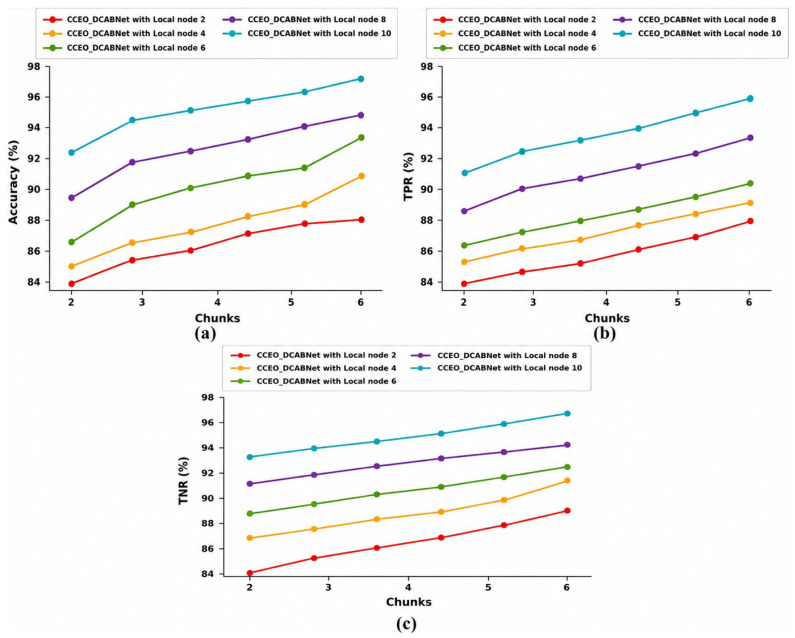
Federated Assessment of CCEO-DCABNet using Pulmonary Edema dataset, (**a**) accuracy, (**b**) TPR, (**c**) TNR.

1. Evaluation using NIH Chest X-ray Dataset
  Figure 12 shows Federated assessment of CCEO-DCABNet using NIH Chest X-ray dataset. Analysis regarding accuracy is deliberated in Figure 12a. Here, CCEO-DCABNet attains an accuracy of 86.32%, 87.63%, 90.78%, 92.11% and 94.78% while local nodes are varied from 2 to 10. Figure 12b displays the evaluation regarding TPR. The TPR attained by CCEO-DCABNet at local node 2 is 85.36%, 4 is 87.21%, 6 is 88.32%, 8 is 91.52%, and 10 is 93.56%. Figure 12c portrays an estimation concerning TNR, and TNRs of 86.98%, 88.45%, 90.02%, 92.61%, and 94.63% are attained by CCEO-DCABNet at local nodes 2–10.
2. Evaluation using Pulmonary Edema dataset
  Federated evaluation of CCEO-DCABNet using the Pulmonary Edema dataset is portrayed in Figure 13. Figure 13a deliberates an evaluation concerning accuracy. At local nodes from 2 to 10, accuracy of 84.32%, 86.41%, 88.74%, 91.45%, and 94.85% are attained by CCEO-DCABNet. Evaluation regarding TPR is deliberated in Figure 13b, where TPR attained by CCEO-DCABNet at local nodes 2–10 are 85.14%, 86.32%, 88.45%, 91.12% and 93.65%. Figure 13c portrays an analysis regarding TNR. Here, CCEO-DCABNet attains TPRs at local node 2 is 86.32%, 4 is 88.48%, 6 is 90.35%, 8 is 92.48%, and 10 is 94.78%.

#### 4.8.2. K-Fold Assessment

K-Fold valuation of CCEO-DCABNet is achieved using the NIH Chest X-ray, and Pulmonary Edema datasets, where K-value of 5 is considered for analysis.

**Figure 14:**
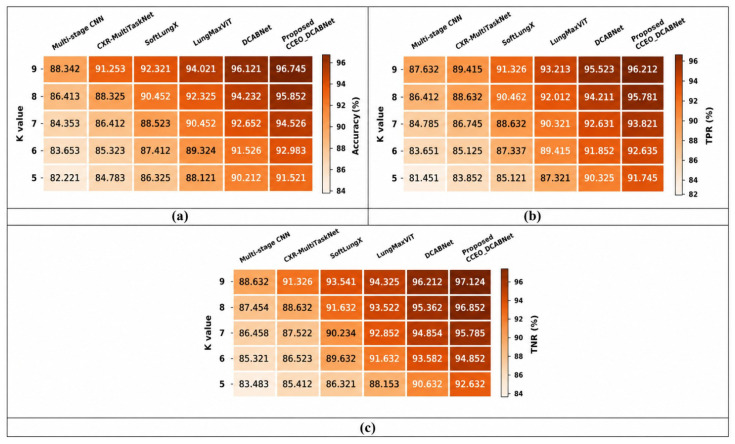
K-Fold Assessment of CCEO-DCABNet using NIH Chest X-ray dataset, (**a**) accuracy, (**b**) TPR, (**c**) TNR.

**Figure 15:**
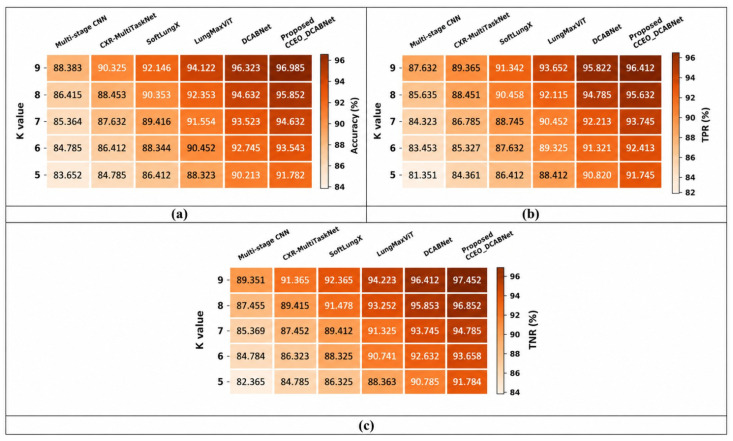
K-Fold Assessment of CCEO-DCABNet using Pulmonary Edema dataset, (**a**) accuracy, (**b**) TPR, (**c**) TNR.

1. Assessment using NIH Chest X-ray Dataset
  Figure 14 deliberates K-Fold valuation of CCEO-DCABNet by NIH Chest X-ray dataset. Assessment regarding accuracy is shown in Figure 14a. Existing models and CCEO-DCABNet attain an accuracy of 82.22%, 84.78%, 86.32%, 88.12%, 90.21% and 91.52%. Thus, CCEO-DCABNet achieves 10.16%, 7.362%, 5.677%, 3.714%, and 1.430% of superior performance than existing models. Figure 14b displays an estimation regarding TPR, where TPR achieved by CCEO-DCABNet is 91.74%, and existing techniques attain the values 81.45%, 83.85%, 85.12%, 87.32% and 90.32%. Hence, CCEO-DCABNet attains 11.22%, 8.602%, 7.219%, 4.821%, and 1.547% of improved performance. Figure 14c portrays an evaluation concerning TNR. Here, existing models, and CCEO-DCABNet attains 83.48%, 85.41%, 86.32%, 88.15%, 90.63%, and 92.63% of TNR. Hence, performance improvement of 9.876%, 7.794%, 6.812%, 4.835%, and 2.158% is achieved by CCEO-DCABNet.
2. Assessment using Pulmonary Edema dataset
  K-Fold evaluation of CCEO-DCABNet using the Pulmonary Edema dataset is illustrated in Figure 15. Figure 15a deliberates an evaluation of accuracy. The CCEO-DCABNet and other methods attain 91.78%, 83.65%, 84.78%, 86.41%, 88.32%, and 90.21% of accuracy. Hence, an improved performance of 8.857%, 7.623%, 5.850%, 3.768% and 1.709% is attained by CCEO-DCABNet than others. Analysis regarding TPR is depicted in Figure 15b, where TPR of 81.35%, 84.36%, 86.41%, 88.41%, 90.42%, and 91.74% are achieved by existing models, and CCEO-DCABNet. As a result, CCEO-DCABNet attains better performance of 11.32%, 8.048%, 5.813%, 3.632%, and 1.008%. Figure 15c displays an estimation concerning TNR, where values achieved by CCEO-DCABNet is 91.78%, and existing models are 82.36%, 84.78%, 86.32%, 88.36%, and 90.78%. Thus, CCEO-DCABNet achieves performance improvement of 10.26%, 7.625%, 5.947%, 3.727% and 1.088% than other methods.

### 4.9. Discussion

Comparative discussion with Wilcoxon-statistic of CCEO-DCABNet is illustrated below.

#### 4.9.1. Comparative Discussion

Table 3 displays comparative discussion of the CCEO-DCABNet dual dataset. The finest values are achieved by CCEO-DCABNet using the Pulmonary Edema dataset, where CCEO-DCABNet attains an accuracy of 96.98%, and existing methods are 88.38%, 90.32%, 92.14%, 94.12%, and 96.32%. TPR of 96.41% is achieved by CCEO-DCABNet, and other models attains 87.63%, 89.36%, 91.34%, 93.65% and 95.82% of TPR. The TNR of 97.45% attained by CCEO-DCABNet, and values 89.35%, 91.36%, 92.36%, 94.22%, and 96.41% are attained by values achieved by other methods. Besides, accuracy, TPR, and TNR of 96.74%, 96.21%, and 97.12% are attained by CCEO-DCABNet using the NIH Chest X-ray dataset. Here, CCEO-DCABNet yields an optimal result owing to its superior stability and processing speed.

#### 4.9.2. Wilcoxon Test

Wilcoxon test analyzes performance improvement of CCEO-DCABNet. The tests are done by K-fold cross-validation with the NIH Chest X-ray and Pulmonary Edema Dataset. Here, a *p*-value below 0.05 offers a statistically significant value, and Wilcoxon test results are given in Table 4. Consider the NIH Chest X-ray dataset, CCEO-DCABNet yields an optimal accuracy compared to Multi-stage CNN (Wilcoxon-statistic = 2.96, *p* = 0.035), CXR-MultiTaskNet (Wilcoxon-statistic = 2.84, *p* = 0.033), SoftLungX (Wilcoxon-statistic = 2.48, *p* = 0.025), LungMaxViT (Wilcoxon-statistic = 2.17, *p* = 0.015) and DCABNet (Wilcoxon-statistic = 2.11, *p* = 0.012). Further, statistically significant accuracy, TPR, and TNR are attained by CCEO-DCABNet using Pulmonary Edema dataset. Furthermore, statistically significant outputs display the effectiveness of CCEO-DCABNet.

#### 4.9.3. Convergence Analysis

**Figure 16:**
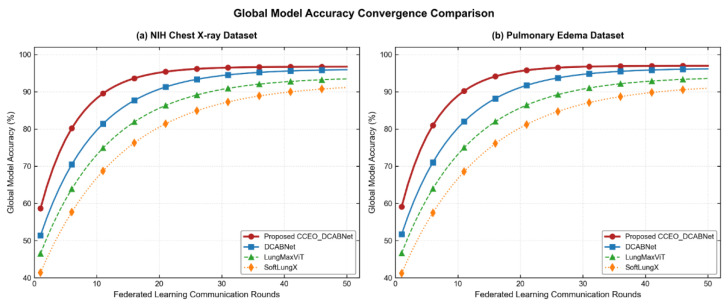
Global model accuracy convergence comparison over 50 communication rounds: (**a**) NIH Chest X-ray dataset, (**b**) Pulmonary Edema dataset.

In order to test the accuracy of the trajectories and training stability of the decentralized architecture, a precise study was conducted on 50 consecutive communication rounds. The global model accuracy trajectories for both the NIH Chest X-ray dataset and the Pulmonary Edema dataset are illustrated in Figure 16. From Figure 16, it is clearly evident that the proposed CCEO-DCABNet framework exhibits rapid learning capability and superior accuracy potential compared with standalone architectures such as DCABNet, LungMaxViT, and SoftLungX. The conventional networks show noticeable fluctuations and slower convergence due to unstable learning under heterogeneous and non-IID data distributions. In contrast, the proposed CCEO optimization strategy enables the global model to converge faster and attain stable accuracy levels of 96.74% on the NIH Chest X-ray dataset and 96.98% on the Pulmonary Edema dataset within approximately 20–25 communication rounds.

**Figure 17:**
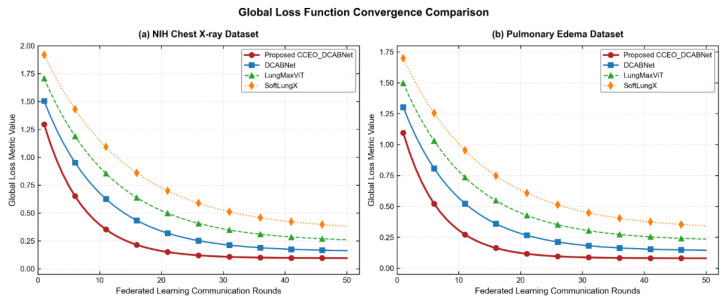
Global loss function convergence profile comparison over 50 federated communication rounds: (**a**) NIH Chest X-ray dataset, (**b**) Pulmonary Edema dataset.

The convergence characteristics are further supported by the global loss profiles shown in Figure 17. As observed in Figure 17a,b, the global cross-entropy loss of the proposed CCEO-DCABNet decreases sharply and converges to lower asymptotic values of 0.095 and 0.080 for the NIH Chest X-ray and Pulmonary Edema datasets, respectively. The loss curves exhibit a smooth descent without irregular oscillations or sudden gradient explosions. From a mathematical perspective, this behavior is attributed to the integration of chaotic evolution optimization (CEO) with chronological memory mechanisms. The chaotic evolution process prevents local nodes from becoming trapped in poor local minima during the early stages of nonlinear boundary optimization, whereas the chronological parameter aggregation at the central server avoids erratic global weight updates. Consequently, the proposed framework demonstrates strong robustness against edge-node heterogeneity and communication latency variations.

### 4.10. Ablation Studies

In order to verify the role and significance of each of the multi-functional modules employed in the suggested approach, an elaborate ablation study was performed. Six different settings for testing the classification framework were examined by gradually eliminating or substituting critical blocks of functionality:

- w/o Image Pre-preparation: Bypassing the initial Gaussian filter-enabled denoising and multiscale unsharp masking-enabled sharpening steps.
- w/o MMPU-Net Segmentation: Removing the automated lung lobe segmentation pipeline, thereby forcing the deep learning streams to process the unsegmented, raw global chest X-ray.
- w/o Radiomic Features: Retaining the segmented regions but completely omitting the extraction of statistical radiomic features (Solidity, Compactness, Roundness, and Eccentricity) from the pipeline.
- w/o Capsule Network: Disabling the deep channel-attention correlative capsule module (DCACorrCapsNet).
- w/o Neural Attention: Isolating the core fusion network from the attention-guided network stream (ADBNet).
- w/o Taylor Fusion (Empirical Concatenation): Replacing the second-order Taylor series expansion mapping with standard empirical feature vector concatenation.
  The quantitative performance metrics across both the NIH Chest X-ray and Pulmonary Edema datasets are compiled in Table 5.

### 4.11. Computational Complexity

The computational overhead of the proposed CCEO-DCABNet framework was measured during the local training and global federation stages:

- Local Latency: Image preprocessing (Gaussian filtering and multiscale unsharp masking) and MMPU-Net segmentation require an average of 4.2 ms and 18.5 ms per chest X-ray, respectively. Computing the explicit 1×4 radiomic vector introduces a negligible latency of <0.8 ms.
- Training and Convergence: Using a local batch size of 32, a single localized training epoch takes approximately 3.2 min. Guided by the CCEO algorithm, local sub-networks reach optimal stability within 25 epochs, while the global federated system achieves complete convergence within 40 to 50 communication rounds.
- Inference Speed: Post-deployment, the end-to-end inference latency for an unseen patient image is 24.5 ms. This near-real-time performance validates the network’s readiness for high-throughput clinical triage.

### 4.12. Clinical Evaluation and Real-World Deployment Scenarios

The CCEO-DCABNet architecture that is presented can facilitate multiclass detection of lung diseases with the help of Chest X-ray images for radiologists as well as other health care experts. With respect to the Federated Learning algorithm adopted, a collaborative model learning process can be initiated within various hospitals without the need to share sensitive information about patients. Moreover, the proposed model can be used as a supplementary support for diagnosing diseases with the help of artificial intelligence to reduce the workload of radiologists and offer an initial analysis. The use of the Grad-CAM algorithm enhances explainability of results by specifying the location of disease in chest X-rays. Direct comparison of the proposed model with radiologists as well as, its deployment in real-time hospitals, will be considered in future work.

## 5. Conclusions

Chest X-ray-based image analysis offers treatment planning for different types of lung illness. Application of FL in the healthcare system provides a better learning from different forms of decentralized data. The FL-based disease classification increases model generalization and also protects patients’ privacy. Still, disease classification is difficult owing to heterogeneous data, overlapping radiographic features, and privacy issues. For solving such issues, a hybrid CCEO-DCABNet-based multiclass disease classification is developed using FL. Developed model attains superior data privacy, where nodes and the server are main entities of FL approach. In the training model, multiclass disease classification is done, where Gaussian filter-enabled denoising and Multiscale Unsharp Masking-enabled sharpening are performed in the pre-preparation phase. Lung lobes are segmented using MMPU-Net, and feature extraction performs an extraction of radiomic features. Moreover, DCABNet performs multiclass disease classification, and it is trained by CCEO. In addition, the average method provides a local update and aggregation process. Furthermore, metrics, like accuracy, TPR, and TNR, estimate the performance of CEO-DCABNet, and yield the values of 96.98%, 96.41%, and 97.45%. In the future, advanced learning techniques will be incorporated to increase data secrecy.

## Figures and Tables

**Table 1:** Dataset partitioning, category distribution, and federated client node allocation matrix.

Simulated Node ID	Target Dataset Source	Normal Class (Images)	Pneumonia Class (Images)	Pleural Effusion Class (Images)	Total Allocated Images
Client Node 1	NIH Chest X-Ray	15,000	2500	3000	20,500
Client Node 2	NIH Chest X-Ray	12,000	4000	2200	18,200
Client Node 3	NIH Chest X-Ray	14,500	1500	4100	20,100
Client Node 4	Pulmonary Edema	300	450 (Alveolar/Interstitial)	250 (Vascular Congestion)	1000
Global Test Set	Combined (20% Holdout)	8360	1690	1910	11,960

**Table 2:** Parameter Details of CCEO.

Parameters	Values
Highest iteration	100
Scaling factor	0.5
Random value	[0, 1]
Dimensions	50
Population dimension	50
Lower bound	−100
Upper bound	100
Learning rate	0.001
Batch size	64
Epochs	100
Kernel size	(5,5)
Padding	Same
Stride	1
Activation (convolution layer)	ReLU
Filter size	64–128
Loss function	Categorical cross-entropy
Dimension of capsules	8
Optimizer	CCEO
Number of capsules	32
Activation (dense layer)	Softmax

**Table 3:** K-fold discussion of CCEO-DCABNet.

Datasets	Metrics/Methods	Multi-Stage CNN	CXR-MultiTask Net	SoftLungX	LungMax ViT	DCAB Net	CCEO_DCABNet
NIH Chest X-ray Dataset	Accuracy (%)	88.34	91.25	92.32	94.02	96.12	96.74
	TPR (%)	87.63	89.41	91.32	93.21	95.52	96.21
	TNR (%)	88.63	91.32	93.54	94.32	96.21	97.12
Pulmonary Edema Dataset	Accuracy (%)	88.38	90.32	92.14	94.12	96.32	96.98
	TPR (%)	87.63	89.36	91.34	93.65	95.82	96.41
	TNR (%)	89.35	91.36	92.36	94.22	96.41	97.45

**Table 4:** Wilcoxon Test results.

Comparative Methods			Multi-Stage CNN	CXR-MultiTaskNet	SoftLungX	LungMaxViT	DCABNet
NIH Chest X-ray Dataset	Wilcoxon-statistic	Accuracy	2.96	2.84	2.48	2.17	2.11
		TPR	2.98	2.89	2.56	2.31	2.22
		TNR	2.92	2.68	2.37	2.12	2.09
	*p*-value	Accuracy	0.035	0.033	0.025	0.015	0.012
		TPR	0.042	0.038	0.031	0.024	0.018
		TNR	0.029	0.029	0.022	0.012	0.009
Pulmonary Edema Dataset	Wilcoxon-Statistic	Accuracy	2.84	2.58	2.29	2.11	2.14
		TPR	2.91	2.69	2.51	2.25	2.28
		TNR	2.75	2.49	2.21	2.04	2.08
	*p*-value	Accuracy	0.031	0.026	0.018	0.012	0.007
		TPR	0.035	0.028	0.022	0.015	0.011
		TNR	0.027	0.021	0.016	0.009	0.005

**Table 5:** Ablation study results of individual structural modules on classification metrics across both datasets.

Model Configuration	Dataset	Accuracy (%)	TPR (%)	TNR (%)
Complete CCEO-DCABNet	NIH Chest X-ray	96.74%	96.21%	97.12%
	Pulmonary Edema	96.98%	96.41%	97.45%
1. w/o Image Pre-preparation	NIH Chest X-ray	91.15%	90.86%	91.50%
	Pulmonary Edema	92.05%	91.12%	92.40%
2. w/o MMPU-Net Segmentation	NIH Chest X-ray	92.30%	91.54%	93.12%
	Pulmonary Edema	91.80%	91.02%	92.65%
3. w/o Radiomic Features	NIH Chest X-ray	93.12%	92.40%	93.75%
	Pulmonary Edema	92.95%	92.10%	93.50%
4. w/o DCACorrCapsNet	NIH Chest X-ray	93.45%	92.90%	94.02%
	Pulmonary Edema	93.10%	92.45%	93.88%
5. w/o ADBNet	NIH Chest X-ray	94.12%	93.55%	94.80%
	Pulmonary Edema	93.95%	93.15%	94.60%
6. w/o Taylor Fusion (Concatenation)	NIH Chest X-ray	90.28%	89.65%	91.10%
	Pulmonary Edema	90.50%	89.92%	91.35%

## Data Availability

Data available on request.
